# Adherent Intestinal Cells From Atlantic Salmon Show Phagocytic Ability and Express Macrophage-Specific Genes

**DOI:** 10.3389/fcell.2020.580848

**Published:** 2020-10-15

**Authors:** Youngjin Park, Qirui Zhang, Geert F. Wiegertjes, Jorge M.O. Fernandes, Viswanath Kiron

**Affiliations:** ^1^Faculty of Biosciences and Aquaculture, Nord University, Bodø, Norway; ^2^Division of Clinical Genetics, Lund University, Lund, Sweden; ^3^Aquaculture and Fisheries Group, Wageningen University & Research, Wageningen, Netherlands

**Keywords:** adherent, intestinal cells, ImageStream^®X^, Atlantic salmon, phagocytosis, RNA-Seq, macrophages

## Abstract

Our knowledge of the intestinal immune system of fish is rather limited compared to mammals. Very little is known about the immune cells including the phagocytic cells in fish intestine. Hence, employing imaging flow cytometry and RNA sequencing, we studied adherent cells isolated from healthy Atlantic salmon. Phagocytic activity and selected gene expression of adherent cells from the distal intestine (adherent intestinal cells, or AIC) were compared with those from head kidney (adherent kidney cells, or AKC). Phagocytic activity of the two cell types was assessed based on the uptake of *Escherichia coli* BioParticles^TM^. AIC showed phagocytic ability but the phagocytes were of different morphology compared to AKC. Transcriptomic analysis revealed that AIC expressed genes associated with macrophages, T cells, and endothelial cells. Heatmap analysis of selected genes indicated that the adherent cells from the two organs had apparently higher expression of macrophage-related genes. We believe that the adherent intestinal cells have phagocytic characteristics and high expression of genes commonly associated with macrophages. We envisage the possibilities for future studies on enriched populations of adherent intestinal cells.

## Introduction

Teleost fishes have both organized and diffuse lymphoid tissues. However, these tissues differ from those of mammals morphologically and functionally. Fish lack bone marrow and lymph nodes found in mammals. Therefore, they rely on thymus, (head) kidney and spleen as their key lymphoid organs. The head kidney (or anterior kidney), is the main source of cytokine-producing lymphoid cells (Geven and Klaren, 2017), macrophages, and plasma cells, which find their way to specific sites, including those in the intestine, to replenish tissue-resident cell populations (Kratofil et al., 2017). Furthermore, the head kidney recruits specific cell types during disease conditions like inflammation (Parra et al., 2015). The head kidney is also a well-known primary B cell organ in fishes (Li et al., 2006; Parra et al., 2015). This makes the head kidney the main hematopoietic organ in teleost fish. In addition, fish possess mucosa-associated lymphoid tissues (MALTs), but these tissues are more diffuse compared to those in mammals (Parra et al., 2015). Among these MALTs, gut-associated lymphoid tissues (GALTs) contain two main populations of immune cells: (1) intraepithelial lymphocytes, which include mainly T cells located between the epithelial cells; and (2) lamina propria leukocytes, which are comprised of lymphocytes, macrophages, granulocytes and plasma cells (Rombout et al., 2011). In-depth knowledge of these immune cells present in GALT is necessary to understand the crosstalk between antigens and the epithelium as well as the immunological functions of both the key lymphoid organs and GALT. However, the lack of appropriate cell markers and complexity of isolation protocols are still hampering the progress of research on leukocyte cell populations in the fish intestine.

The most common practice is to collect adherent cells from specific organs in order to characterize them through further analysis. Cell adhesion refers to the ability of cells to adhere to other cells or extracellular matrix (Khalili and Ahmad, 2015). This process not only stimulates communication between cells but also helps to retain the tissue structure and functions (Khalili and Ahmad, 2015). The cells isolated from tissues or organs are mostly adherent types, and the known mammalian adherent cells are macrophages (Selvarajan et al., 2011), T cells (Bierer and Burakoff, 1988; Shimizu et al., 1991), endothelial cells (Braniste et al., 2016), epithelial cells (Kihara et al., 2018) and dendritic cells (DC, only upon antigen exposure) (Yi and Lu, 2012). Mammalian cell culture methods are frequently adopted to culture and study monocyte-derived macrophage-like cells from fish immunological tissues such as spleen (Iliev et al., 2013) and head kidney (Joerink et al., 2006; Iliev et al., 2019). However, our knowledge of the cell types including phagocytic cells that are involved in the fish intestinal immune response is rather limited compared to mammals.

As for the intestinal cells of fish, McMillan and Secombes (1997) were the first researchers to describe protocols to isolate them from rainbow trout, *Oncorhynchus mykiss*. After a decade, several research groups put in effort to effectively isolate and characterize intestinal immune cells from different fish species such as rainbow trout (Bernard et al., 2006), gilthead seabream, *Sparus aurata* (Salinas et al., 2007) and Atlantic salmon, *Salmo salar* (Attaya et al., 2018). Although there is ample information about the adherent cells from the head kidney in teleost fishes, knowledge about the intestinal cells has to be gathered by employing next generation techniques.

Therefore, this study investigated the adherent cells isolated from the distal intestine by employing the cells from the head kidney of Atlantic salmon as reference. We adopted two high-throughput techniques, imaging flow cytometry (IFC) and RNA sequencing. First, using IFC we explored the adherent cells from the aforementioned organs to decipher the characteristics of the populations and their phagocytic activity. Subsequently, a transcriptomic study was carried out to profile the expression of (1) cell type (macrophage, dendritic cell, endothelial cell, T and B cells)-related genes and (2) other immune genes (cytokines, chemokines, mucins and toll-like receptors) to delineate if the adherent cells expressed genes associated with phagocytes.

## Materials and Methods

### Experimental Fish and Sampling Procedure

Atlantic salmon post smolts of about 70 g were purchased from a commercial producer (Sundsfjord Smolt, Nygårdsjøen, Norway) and maintained at the Research Station of Nord University, Bodø, Norway. Fish were fed a commercial feed (Ewos Micro, Ewos AS, Bergen, Norway) to satiation, and reared in a flow-through sea water system (temperature: 7–8°C, dissolved oxygen saturation: 87–92%, 24-h light cycle). Fish (of the weight range 510–590 g) was used in this study. The fish were starved for 24 h and were killed with an over dose of MS-222 (Tricaine methane sulfonate; Argent Chemical Laboratories, Redmond, United States; 80 mg/L). Head kidney (HK) and distal intestine (DI) samples were then collected.

### Cell Isolation and Culture

Immune cells from the head kidney (HK) and distal intestine (DI) were isolated and grown at 12°C in Leibovitz’s L-15 Medium (L-15; Sigma-Aldrich, Oslo, Norway) as described previously by Park et al. (2020) and Salinas et al. (2007), respectively. The osmolality of cell culture media was adjusted to 380 mOsm by adding a solution consisting of 5% (v/v) 0.41 M NaCl, 0.33 M NaHCO_3_ and 0.66 (w/v) D-glucose (Sigma).

Briefly, HK from salmon (*n* = 6) were sampled and transferred to 15 mL centrifuge tubes to make a total volume of 4 mL in ice-cold L-15 + [L-15 medium with 50 U/mL penicillin (Sigma), 50 μg/mL streptomycin (Sigma), 2% fetal bovine serum (FBS; Sigma) and 10 U/mL heparin (Sigma)]. The tissues were passed through a sterile 100-μM cell strainer (Falcon, New York, United States) with ice-cold L-15 +. Thereafter, the cell suspensions were layered on 40%/60% Percoll (Sigma) to separate HK leukocytes and centrifuged at 500 × *g* for 30 min, at 4°C. Cells at the interface were collected and washed twice with ice-cold L-15-FBS free (L-15 medium with 50 U/mL penicillin, 50 μg/mL streptomycin) by centrifugation (500 × *g*, 5 min, 4°C).

For intestinal cell isolation, DI samples from salmon (*n* = 6) were transferred to a cell culture dish (Nunc EasyDish, Thermo Fisher Scientific, Oslo, Norway) with 2 mL ice-cold PBS. The tissues were cut open longitudinally and washed with ice-cold PBS to remove gut contents. After washing they were cut into small pieces (1–2 cm fragments) and transferred to 15 mL centrifuge tubes to make a total volume of 4 mL in DTT solution (0.145 mg/mL dithiothreitol + 0.37 mg/mL EDTA in Ca^2+^ and Mg^2+^ free HBSS, Sigma) at room temperature for 20 min to break disulfide bonds in the mucus. Next, the tissue fragments were washed with L-15 + supplemented with DNAse (0.05 mg/ml; Sigma) to prevent cell clumping and wash out excess DTT. Thereafter, the washed fragments were transferred to 15 mL centrifuge tubes to make a total volume of 6 mL in the digestive solution (0.37 mg/mL collagenase IV, Thermo Fisher Scientific). The centrifuge tubes were incubated in a shaking incubator (200 rpm) at RT for 60 min. The tissue fragments and supernatants were then passed through a sterile 100-μM cell strainer with ice-cold L-15 +, and the cell suspensions were layered on 25%/75% Percoll. The tubes containing the cells and Percoll were centrifuged at 500 × *g* for 30 min, at 4°C to separate DI leukocytes. Cells at the interface were collected and washed twice with ice-cold L-15-FBS free by centrifugation (500 × *g*, 5 min, 4°C).

Both HK and DI leukocytes (mentioned under flow cytometry studies) were allowed to adhere on a cell culture dish with 2 mL L-15 + for 2 days at 12°C. After collecting the supernatant containing non-adherent cells, the dish with the adherent cells was placed on ice for 10 min, and the cells were detached by washing three times with 1.5 mL ice-cold PBS supplemented with 5 mM EDTA. The cells obtained were centrifuged (500 × *g*, 5 min, 4°C) and re-suspended in 1 mL L-15 +. Then, the cells were counted using a portable cell counter (Scepter 2.0 cell counter, EMD Millipore, Darmstadt, Germany). To confirm the quality of the harvested cells, we observed the cells using a live cell imager (ZOE Fluorescent Cell imager, Bio-Rad, Oslo, Norway). With our improved protocols, we were able to harvest many cells with high viability. We checked the quality of the isolated cells by observing them through microscope and with the help of live/dead cell assays using propidium iodide.

### Flow Cytometry Studies

To understand the cell populations and their functions we isolated and cultured cells from HK and DI. These cells were studied employing the ImageStream^®X^ Mk II Imaging Flow Cytometer (Luminex Corporation, Austin, TX, United States) equipped with two argon-ion lasers (488 and 642 nm) and a side scatter laser (785 nm). The acquired cell data were analyzed using IDEAS 6.1.822.0 software (Luminex). All flow cytometry assays were performed as described previously (Park et al., 2020).

#### Cell Population

To study the cell populations − (i) whole leukocytes, (ii) supernatants and (iii) adherent cells − from three cell suspensions from HK or DI, aliquots containing > 5 × 10^5^ cells were washed with 500 μL PBS by centrifugation (500 × *g*, 5 min, 4°C) and resuspended in 50 μL PBS. Prior to loading the tubes containing the cells in the flow cytometer, 1 μL of propidium iodide (PI, 1 mg/mL, Sigma) was added to differentiate between the dead and live cells as well as to estimate the cell types based on the morphology of their nucleus. From each sample, more than 10,000 cell images were acquired using two lasers with optimized voltage levels, 488 nm (1 mW) and 785 nm (0.47 mW). Thereafter, using IDEAS software, dead cells were excluded based on PI positivity; both are shown in a brightfield (BF) area (cell size) vs. side scatter (SSC) intensity (cell granularity) plot.

#### Phagocytosis Assay

To compare the phagocytic activity of whole leukocytes and adherent cells (i and iii mentioned under flow cytometry studies), aliquots containing 0.5 × 10^5^ cells in 100 μL L-15 + were incubated with fluorescent bio-particles (pHrodo^TM^ Red *Escherichia coli* Bioparticles, Thermo Fisher Scientific), at a cell and particle ratio of 1:5 for 2 h at 12°C. After incubation, the cells were washed once with 500 μL PBS by centrifugation (500 × *g*, 5 min, 4°C) and resuspended in 50 μL PBS. Thereafter, the tubes containing cells were loaded in the flow cytometer and more than 10,000 cell images were acquired using two lasers with optimized voltage levels, 488 nm (50 mW) and 785 nm (0.47 mW). Data analyses were performed following our previous protocol (Park et al., 2020). Phagocytic ability was measured as the percent of phagocytic cells in total cells while phagocytic capacity was calculated as the mean number of particles in each phagocyte as described previously (Park et al., 2020).

### Transcriptomic Analysis

The main aim of the RNA-Seq analysis was to obtain a snapshot of the expression profiles of selected genes. To compare the expression of genes linked to the adherent cells from HK and DI, 12 libraries were prepared (*n* = 6). The list of selected genes used in this study is comprised of (1) 34 cell type (macrophage, dendritic cell, endothelial cell, T and B cells)-related genes and (2) 42 other immune-related (cytokines, chemokines, mucins and toll-like receptors) genes, as shown in Tables 1 and 2, respectively.

**TABLE 1:** List of abbreviations and details of genes expressed in macrophages, dendritic cells, endothelial cells, T and B cells.

**Abbreviations**	**Gene name**	**Ensembl/GenBank ID**
*cd68*	CD68	ENSSSAG00000002993
*cd200r1*	CD200 receptor 1A-like	ENSSSAG00000039924
*mmd*	monocyte to macrophage differentiation protein	ENSSSAG00000001828
*csf1r*	macrophage receptor MARCO-like	ENSSSAG00000063051
*marco*	macrophage colony stimulating factor receptor-like protein	ENSSSAG00000061479
*mpeg1*	macrophage expressed 1	ENSSSAG00000076214
*capg*	macrophage-capping protein-like	ENSSSAG00000003660
*h2-eb1*	H-2 class II histocompatibility antigen, I-E beta chain	XM_014133067
*cd74*	HLA class II histocompatibility antigen gamma chain-like	ENSSSAG00000004635
*cd80*	CD80-like	ENSSSAG00000056420
*cd83*	CD83 antigen	ENSSSAG00000057240
*cd209*	CD209 antigen-like	XM_014194638
*cd3gda*	CD3gammadelta-A	ENSSSAG00000009995
*cd3z*	CD3zeta-1	ENSSSAG00000055061
*cd42al*	truncated CD4-2A-like protein	ENSSSAG00000076595
*cd8a*	CD8 alpha	ENSSSAG00000065860
*cd8b*	CD8 beta	ENSSSAG00000045680
*cd28*	T-cell-specific surface glycoprotein CD28-like	ENSSSAG00000060163
*cd2l*	T-cell surface antigen CD2-like	XM_014129565
*cd96*	T-cell surface protein tactile-like	XM_014129863
*trbc1*	T-cell receptor beta-1 chain C region-like	NC_027300
*trgc2*	T-cell receptor gamma chain C region C10.5-like	NC_027319
*cd6l*	T-cell differentiation antigen CD6-like	ENSSSAG00000057293
*tcd80l*	T-lymphocyte activation antigen CD80-like	ENSSSAG00000056420
*tagap*	T-cell activation GTPase activating protein	ENSSSAG00000061533
*igm*	IgM	XM_014203125
*cd48l*	CD48 antigen-like	ENSSSAG00000064252
*cd79a*	B-cell antigen receptor complex-associated protein alpha chain-like	ENSSSAG00000003014
*blnk*	B-cell linker	ENSSSAG00000065613
*bcap29*	B cell receptor associated protein 29	ENSSSAG00000074312
*bcl9l*	B-cell CLL/lymphoma 9 protein-like	ENSSSAG00000063108
*ecscr*	endothelial cell-specific chemotaxis regulator-like	XM_014129374
*cd151al*	CD151 antigen-like	ENSSSAG00000065694
*pecam*	platelet endothelial cell adhesion molecule-like	ENSSSAG00000001458

**TABLE 2:** List of abbreviations and details of genes coding for selected cytokines, chemokines, mucins, and toll-like receptors.

**Abbreviations**	**Gene name**	**Ensembl/GenBank ID**
*tgfbr1*	Transforming growth factor beta receptor type-1-like	ENSSSAG00000079756
*tgfbr2*	Transforming growth factor beta receptor type-2	ENSSSAG00000071570
*tab2*	Transforming growth factor beta-activated kinase 1 and MAP3K7-binding protein 2-like	ENSSSAG00000074549
*traf2*	Tumor necrosis factor receptor associated factor 2	ENSSSAG00000046864
*tnfa2*	Tumor necrosis factor alpha-2 precursor	ENSSSAG00000053783
*tnfaip2*	Tumor necrosis factor alpha-induced protein 2-like	XM_014143804
*tnfrsf6b*	Tumor necrosis factor receptor superfamily member 6b	ENSSSAG00000044783
*tnfrsf10a*	Tumor necrosis factor receptor superfamily member 10A-like	ENSSSAG00000049387
*tnfsf11*	Tumor necrosis factor superfamily member 11	ENSSSAG00000053734
*tnfrsf13b*	Tumor necrosis factor receptor superfamily member 13B-like	ENSSSAG00000079103
*il1b-like*	Interleukin-1 beta-like	ENSSSAG00000060993
*irak3-like*	Interleukin-1 receptor-associated kinase 3-like	ENSSSAG00000051931
*il1rl1*	Interleukin-1 receptor-like protein	ENSSSAG00000069876
*il1rap*	Interleukin 1 receptor accessory protein	ENSSSAG00000043510
*il6r*	Interleukin-6 receptor subunit alpha	ENSSSAG00000052467
*il6rb*	Interleukin-6 receptor subunit beta-like	ENSSSAG00000061284
*il8*	Interleukin 8	ENSSSAG00000006498
*il12rb2*	Interleukin-12 receptor beta-2 chain	ENSSSAG00000042273
*il17rcl*	Interleukin-17 receptor C-like	ENSSSAG00000007189
*il17rel*	Interleukin-17 receptor E-like	XM_014134812
*il31ra*	Interleukin-31 receptor A	ENSSSAG00000062925
*cxcr1l*	C-X-C chemokine receptor type 1-like	ENSSSAG00000042001
*ccl2l*	C-C motif chemokine 2-like	XM_014191804
*cxcr3l*	C-X-C chemokine receptor type 3-like	ENSSSAG00000051147
*ccr3*	C-C chemokine receptor type 3	ENSSSAG00000042484
*cxcr4l*	C-X-C chemokine receptor type 4-like	ENSSSAG00000050781
*cxcr5l*	C-X-C chemokine receptor type 5-like	ENSSSAG00000040121
*ccr5l*	C-C chemokine receptor type 5-like	ENSSSAG00000072678
*ccr6l*	C-C chemokine receptor type 6	ENSSSAG00000076547
*ccr9l*	C-C chemokine receptor type 9-like	ENSSSAG00000070198
*ccl19l*	C-C motif chemokine 19-like	ENSSSAG00000002773
*ccl20l*	C-C motif chemokine 20-like	ENSSSAG00000051350
*ccl25*	C-C motif chemokine 25	ENSSSAG00000071212
*muc1*	mucin 1, cell surface associated	XM_014160723
*muc5acl*	Mucin-5AC-like	ENSSSAG00000080927
*muc13l*	Mucin-13-like	ENSSSAG00000069082
*muc17l*	Mucin-17-like	ENSSSAG00000047784
*cd164l2*	CD164 sialomucin-like 2 protein	XM_014143111
*tlr2*	Toll-like receptor 2	ENSSSAG00000003781
*tlr6*	Toll-like receptor 6	ENSSSAG00000079217
*tlr8*	Toll-like receptor 8	ENSSSAG00000074528
*tlr13*	Toll-like receptor 13	ENSSSAG00000077966

### RNA Isolation, Library Preparation and Illumina Sequencing

Total RNA was extracted from the adherent cells that were isolated from HK and DI (<500,000 cells) using the PicoPure RNA isolation kit (Thermo Fisher Scientific) according to the manufacturer’s protocol. The quality and quantity of total RNA were assessed using Agilent RNA high sensitivity screen tape kits and Bioanalyzer 2200 TapeStation system (Agilent Technologies, Santa Clara, CA, United States). RNA sequencing libraries were prepared using the NEBNext Ultra II Directional RNA library preparation kit with poly (A) mRNA magnetic isolation module (NEB #E7490; New England BioLabs^®^, Herts, United Kingdom), according to the manufacturer’s protocol. For the first and second strand cDNA synthesis, 50 ng of total RNA having high RNA integrity number (RIN > 8) was enriched with Oligo(dT)-conjugated magnetic beads and fragmented to ∼200 nt. Thereafter, the resulting cDNA were end-repaired for adaptor ligation. The ligated cDNAs were amplified with barcoded primers on a thermal cycler for 14 cycles. The PCR products were purified using AMPure XP beads (Beckman Coulter Inc., Brea, CA, United States) to avoid contamination from residual adapter dimers and unwanted (smaller) fragments. After library preparation, the quality and quantity of individual libraries were assessed using Agilent DNA high sensitivity screen tape kits and Bioanalyzer 2200 TapeStation system. These barcoded individual libraries were pooled at equimolar ratios and sequenced as single−end reads (75 bp) on an Illumina NextSeq 500 sequencer (Illumina, San Diego, CA, United States) with NextSeq 500/550 high output v2 reagents kit (Illumina) at the sequencing facility of Nord University, Bodø, Norway.

### Bioinformatics Analyses

All bioinformatics analyses of RNA-Seq data were performed as described previously by Zhang et al. (2018). The obtained raw sequencing data was deposited in the Gene Expression Omnibus (GEO, NCBI); the accession number is GSE154142. Briefly, raw sequence data were converted to FASTQ format with bcl2fastq2 (v2.17, Illumina). The adapter sequences were removed using cutadapt (version 1.12) with the following the parameters: -q 20 –quality-base = 33 -m 20 –trim-n. The quality of the trimmed fastq files (clean reads) was then assessed using FastQC (Andrews, 2010), and the reads with quality > Q30 were mapped to the Atlantic salmon genome ICSASG_v2 from RefSeq^1^. The mapped reads were quantified and then annotated using gff3 annotation file. The generated data was normalized using DESeq2 (Love et al., 2014), and the normalized data was used for statistical comparisons, i.e., to determine the differences in the expression of selected immune-related genes in AIC and AKC. The package, DESeq2 employs shrinkage estimation for dispersions and fold changes. The R packages ggplot2 version 3.1.1 (Wickham, 2016) were employed to prepare and format the graphs. A heatmap was prepared using the functions from the package ComplexHeatmap (Gu, 2015).

### Statistical Analyses

Statistical analysis was performed using RStudio version 1.1.463. Normality of the flow cytometry and gene expression data was tested by Shapiro-Wilk Test, and the assumption of equal variance was checked by Bartlett’s Test. Comparisons between groups were performed using unpaired Student’s *t*-test. The differences were considered significant at *p* < 0.05.

## Results

### Diverse Cells Among Salmon Distal Intestine Cell Population

**FIGURE 1:**
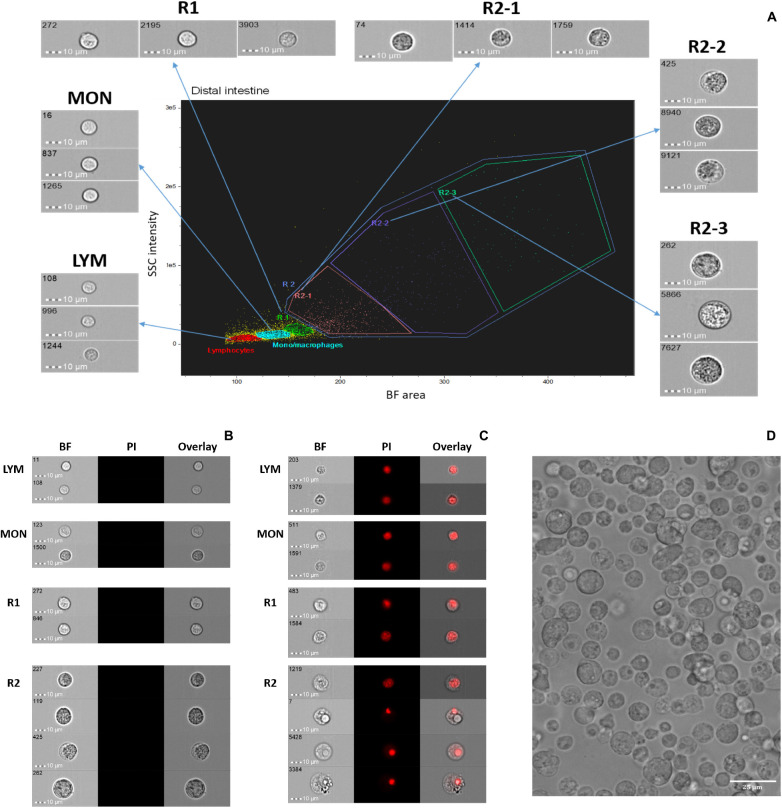
Cell populations from Atlantic salmon distal intestine. **(A)** Live cells are shown in a brightfield (BF) area (cell size) vs. side scatter (SSC) intensity plot (cell granularity). Representative images (40 × objective, scale bar = 10 μm) from each gate are shown separately. Intestinal cells were identified based on nucleus morphologies. Using propidium iodide, live or no-stained cells **(B)** and dead or nucleus-stained cells **(C)** are shown. **(D)** A representative image captured by fluorescent imager (20 × objective, scale bar = 25 μm) shows salmon intestinal cell population. LYM, lymphocytes; MON, monocytes/macrophages; R, region: R1, macrophage-like cells; R2, unknown cells; R2-1, R2-2, presumptive granulocytes; R2-3, presumptive vacuolated cells.

We explored the cell populations from salmon DI by employing IFC. After dead cell exclusion, approximately 90% of viable cells (negative for PI) were obtained; shown in a BF area (cell size) vs. SSC intensity (cell granularity) plot (Figure 1A). Because we have already revealed the identity of the HK cell population in our previous study (Park et al., 2020) here we show only the DI cell population in Figure 1. The gating strategy that was developed previously by Park et al. (2020) was employed for DI cell populations (Figure 1A: LYM, lymphocyte; MON, monocyte/macrophage; R1, macrophage-like cells). Nucleus morphologies were revealed through PI staining (Figures 1B,C). Only PI negative live cells (Figure 1B) were considered for the gating shown in Figure 1A; cells in the gates LYM, MON, and R1 had spherical and fairly rigid nuclei while those in the gate R2 had relatively smaller and different shaped nuclei. We found that there are diverse cells in the isolated salmon DI cell population. A representative image of the DI cell images from among 6 images captured (to confirm the quality of the harvested DI cells) by the fluorescent imager is shown in Figure 1D.

### Adherent Cells From Salmon Distal Intestine Exhibit Phagocytic Ability

**FIGURE 2:**
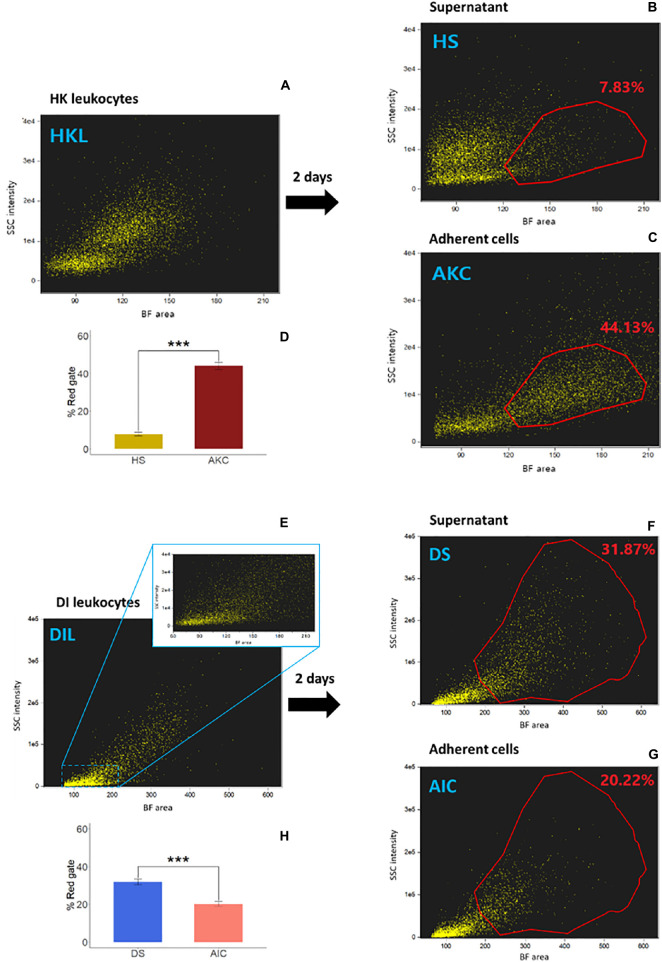
Examination of adherent cell population from Atlantic salmon head kidney and distal intestine by imaging flow cytometry. We compared cell populations from three cell suspensions; (**A** and **E**) whole leukocytes (HKL and DIL, respectively), (**B** and **F**) supernatants (HS and DS, respectively) and (**C** and **G**) adherent cells (AKC and AIC, respectively) in a brightfield (BF) area (cell size) vs. side scatter (SSC) intensity plot (cell granularity) using IFC. The bar plots (**D** and **H**) indicate percent of the cells in the red gates in supernatants (**B** and **F**) vs. those in adherent cells (**C** and **G**, respectively). Statistically significant differences (*p* < 0.001) are indicated using asterisks. Bar plots show the mean ± SD (*n* = 6).

The cell populations − whole leukocytes (HKL and DIL, respectively), supernatants (HS and DS, respectively) and adherent cells (AKC and AIC, respectively) − from three cell suspensions from HK or DI are shown in Figures 2A–C and Figures 2E–G, respectively. The reference AKC had macrophage population, located in higher BF area and higher SSC intensity compared to those of head kidney supernatant-derived cells (HS, red gates; Figures 2B–D). In the DI cells, the cell populations were more scattered, as evident in the dotplot (considering the BF area values). However, AIC apparently had a lower percentage of the cells in the gated area compared to those of distal intestine supernatant-derived cells (DS, red gates; Figures 2F–H).

**FIGURE 3:**
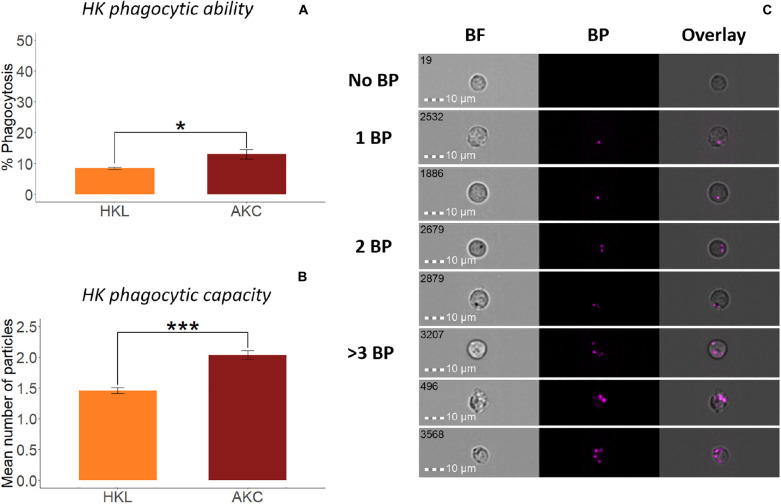
Phagocytic activity of whole leukocytes and adherent cells from Atlantic salmon head kidney. Percent of phagocytic cells or phagocytic ability **(A)** and mean number of bio-particles (BP) ingested per phagocytic cell or phagocytic capacity **(B)** are shown. **(C)** Representative cell images show cells with no BP, and 1BP, 2BP, and >3BP. Statistically significant differences (*p* < 0.05) are indicated using asterisks. Bar plots show the mean ± SD (*n* = 6). All cell images were captured with 40 × objective. Scale bar = 10 μm. BF, brightfield; 1 BP, 2 BP, and > 3 BP, 1–3 internalized bio-particles.

**FIGURE 4:**
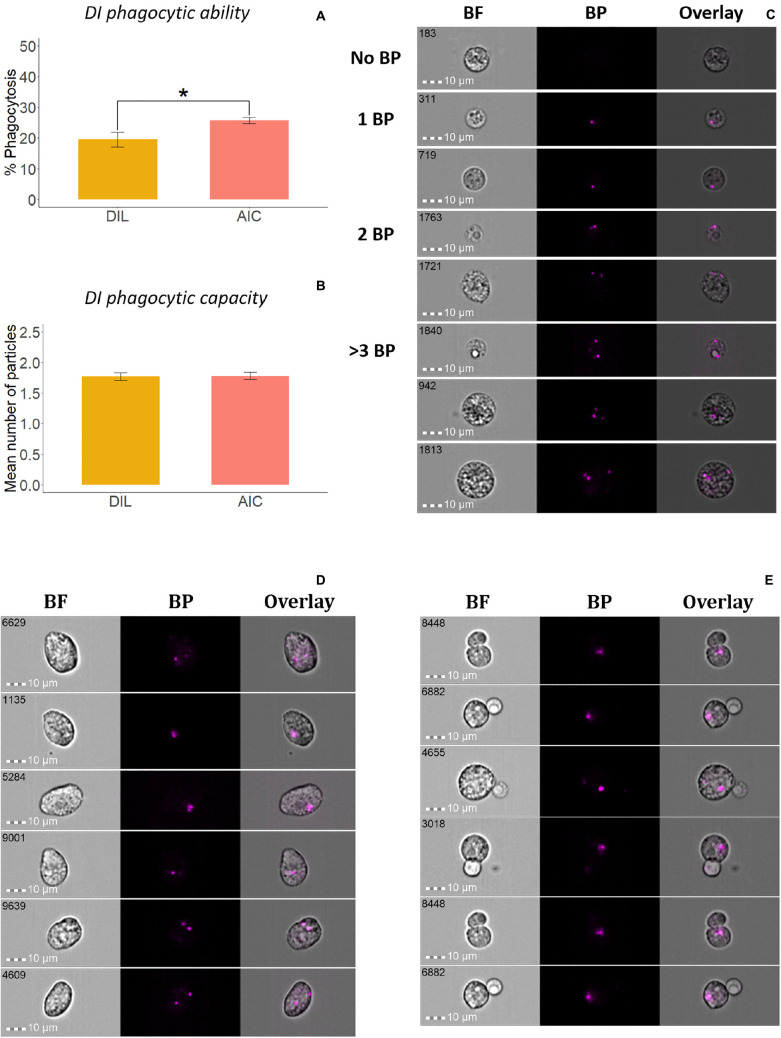
Phagocytic activity of whole leukocytes and adherent cells from Atlantic salmon distal intestine. Percent of phagocytic cells or phagocytic ability **(A)** and mean number of bio-particles (BP) ingested per phagocytic cell or phagocytic capacity **(B)** are shown. **(C)** Representative cell images show cells with no BP, and 1BP, 2BP, and >3BP. Statistically significant differences (*p* < 0.05) are indicated using asterisks. In addition, oval shaped phagocytes **(D)** and doublets containing a phagocyte that interacts with a lymphocyte-like cell **(E)** were found among the adherent intestinal cells. Bar plots show the mean ± SD (*n* = 6). All cell images were captured with 40 × objective. Scale bar = 10 μm. BF, brightfield; 1 BP, 2 BP, and >3 BP, 1–3 internalized bio-particles.

To compare the phagocytic activity of whole leukocytes and adherent cells from HK (Figures 3A–C) and DI (Figures 4A–C), we quantified the uptake of *E. coli* BioParticles^TM^ by the cells (Park et al., 2020). The phagocytic abilities of AKC and AIC were significantly higher than those of HKL and DIL (Figures 3A, 4A), respectively. The phagocytes from AIC had different morphologies compared to AKC (Figures 3C, 4C). Among AICs, along with single and round shaped phagocytes, we found oval shaped (5.69% of total phagocytes, Figure 4D) and doublets consisting of a phagocytic cell and an interacting cell (1.15% of total phagocytes, Figure 4E).

### Adherent Cells From Distal Intestine of Salmon Express Macrophage and Immune-Related Genes

To understand the identity of cell types in AIC, the expression of selected genes in AIC was compared with those of AKC. We obtained over 312 million cleaned reads from 12 (6 from AIC and 6 from AKC) samples after adapter trimming and quality filtering. Of them, over 285 million reads were mapped to Atlantic salmon genome. Average mapping percentage among samples was 91.36% (Supplementary Table 1). The dispersion of the genes decreased with increase in mean of normalized counts (Supplementary Figure 1).

Employing the normalized read counts from DESeq2 analyses, we describe the expression of 34 cell-specific (macrophage, dendritic cell, endothelial cell, T and B cells) genes and 42 immune-related (cytokines, chemokines, mucins and toll-like receptors) genes in AIC compared to AKC. Lack of appropriate cell markers and unavailability of easy intestinal cell isolation techniques hamper the characterization of adherent cells. Therefore, we adopted the targeted analysis strategy to delineate the cell types by linking them to known genes.

**FIGURE 5:**
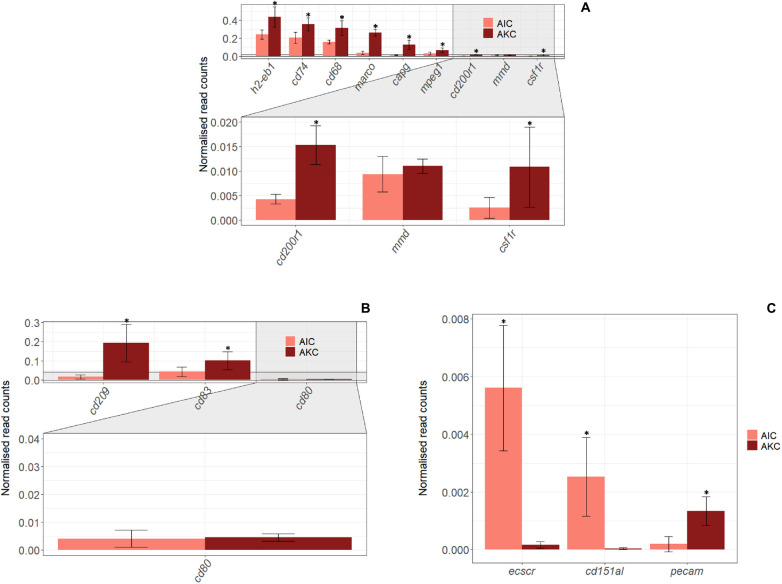
The expression of genes linked to macrophages, dendritic cells and endothelial cells in the adherent cells from Atlantic salmon distal intestine and head kidney. Employing the normalized read counts from DESeq2 analyses, the expression of genes related to macrophages **(A)**, dendritic cells **(B)** and endothelial cells **(C)** were determined, and the expression levels between the adherent cells from salmon distal intestine (AIC) and head kidney (AKC) were compared. Statistically significant differences (*p* < 0.05) are indicated using asterisks. Bar plots show the mean ± SD. Sample size = 6.

**FIGURE 6:**
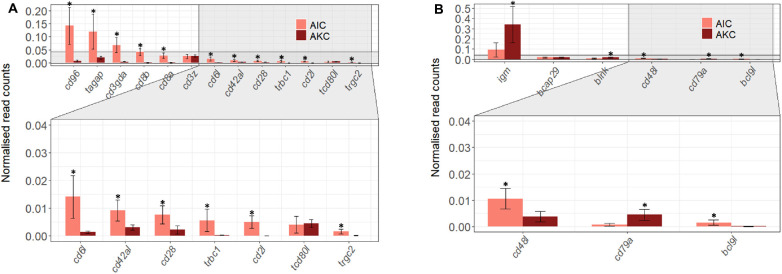
The expression of genes related to T and B cells in the adherent cells from Atlantic salmon distal intestine and head kidney. Employing the normalized read counts from DESeq2 analyses, the expression of T **(A)** and B cells **(B)** associated genes in the adherent cells from salmon distal intestine (AIC) and head kidney (AKC) were compared. Statistically significant differences (*p* < 0.05) are indicated using asterisks. Bar plots show the mean ± SD (*n* = 6).

The expression of 34 genes associated with macrophages, dendritic cells (DC), endothelial cells, T and B cells in AIC were compared to those in AKC (Figures 5, 6). Among the 9 macrophage-related genes, the expression of 8 genes (*h2-eb1*, *cd74*, *cd68*, *marco*, *capg*, *mpeg1*, *cd200r1*, and *csf1r*) was significantly lower in AIC compared to AKC (Figure 5A). As for the DC specific genes (Figure 5B), AIC had significantly lower expression of *cd209* and *cd83* than those of AKC. As for the endothelial cell-related genes, the expression of *ecscr* and *cd151l* was significantly higher in AIC than in AKC though this was not the case for the *pecam* gene (Figure 5C).

Regarding the T and B cell-specific gene profile (Figure 6), AIC had significantly higher expression of most T cell-related genes (*cd96*, *tagap*, *cd3gda*, *cd8b*, *cd8a*, *cd6l*, *cd42al*, *cd28*, *trbc1*, *cd2l*, and *trgc2*), but significantly lower expression of B cell-related genes (*igm*, *blnk*, and *cd79a*).

**FIGURE 7:**
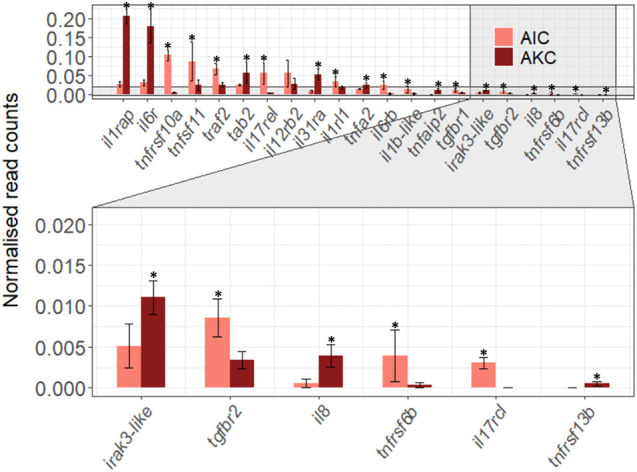
The expression of cytokine-related genes in the adherent cells from Atlantic salmon distal intestine and head kidney. Employing the normalized read counts from DESeq2 analyses, the expression of cytokine-related genes in the adherent cells from salmon distal intestine (AIC) and head kidney (AKC) were compared. Statistically significant differences (*p* < 0.05) are indicated using asterisks. Bar plots show the mean ± SD (*n* = 6).

The expression of 21 immune-related cytokine genes were also considered for the AIC vs. AKC comparisons (Figure 7). AIC had significantly lower expression of interleukin 1 and 6 receptors (*il1rap* and *il6r*) while the expression of tumor necrosis factor (TNF)-related genes (*tnfrsf10a*, *tnfsf11, traf2*, and *tnfrsf6b*) was significantly higher. Among the transforming growth factor (TGF)-related genes, the expression of TGFβ receptors (*tgfbr1* and *tgfbr2*) were significantly higher, while the expression of TGFβ activated kinase binding protein (*tab2*) was significantly lower in AIC.

**FIGURE 8:**
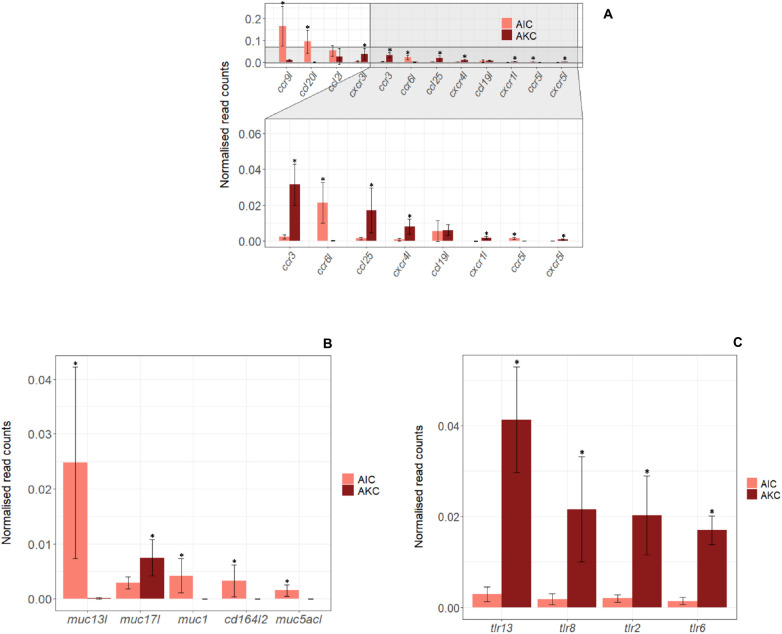
The expression of genes related to chemokines, mucins and toll-like receptors in the adherent cells from Atlantic salmon distal intestine and head kidney. Employing the normalized read counts from DESeq2 analyses, the expression of genes related to chemokines **(A)**, mucins **(B)** and toll-like receptors **(C)** in the adherent cells from salmon distal intestine (AIC) and head kidney (AKC) were compared. Statistically significant differences (*p* < 0.05) are indicated using asterisks. Bar plots show the mean ± SD (*n* = 6).

Twelve immune-related genes of chemokines were also studied in AIC and AKC (Figure 8A). The expression levels of *ccr9l*, *ccl20l*, *ccr6l*, and *ccr5l* were significantly higher, while the expression of, *cxcr3l*, *ccr3*, *ccl25*, *cxcr4l*, *cxcr1l*, and *cxcr5l* was significantly lower in AIC.

The expression of 9 immune-related genes for mucin and toll-like receptor (TLR) were also compared to understand the differences in AIC and AKC (Figures 8B,C). Mucin-related genes (*muc13l*, *muc1*, *cd164l2*, and *muc5acl*) were mainly expressed in AIC but several TLR genes (*tlr13*, *tlr8*, *tlr2* and *tlr6*) were not predominantly expressed in AIC.

**FIGURE 9:**
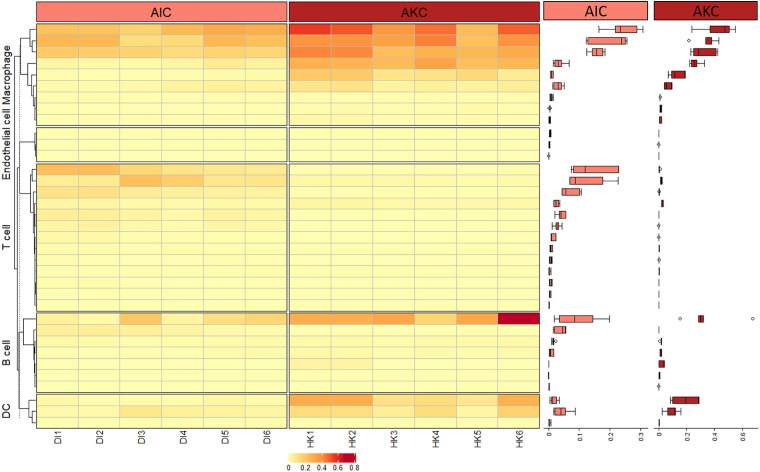
Heatmap of the expression of selected genes in the adherent cells from Atlantic salmon distal intestine and head kidney. Box plots indicate the expression of genes related to macrophages, dendritic cells, endothelial cells, T and B cells. The color gradient from light yellow to dark red in the heatmap indicates increasing transcript levels.

Hierarchical clustering-based heatmap along with the boxplot annotation shown in Figure 9 reveals the gene profiles associated with AIC and AKC. Although the expression of the genes associated with AKC macrophages was significantly higher than those of AIC, both showed predominantly higher expression of the macrophage-specific genes than those of other cell types such as T and B cells.

## Discussion

The intestine has a key role in sustaining the health of fishes. Various cells in the intestine act in harmony to maintain the local (Okumura and Takeda, 2016) as well as systemic homeostasis (Biteau et al., 2011; Ramakrishnan et al., 2019). Of these cells, the GALT cells are imperative in responding to antigens, both benign and pathogenic. The immune cells that are developed in the main lymphoid site enter the circulation, and along with the GALT cells they surveil the host immune status. In the present study, we obtained leukocytes from DI and HK of Atlantic salmon and then separated the adherent cells from the two cell populations.

Employing IFC, we examined the phagocytic activity of the adherent intestine cells and compared it with those of adherent head kidney cells. We also examined the expression of immunologically relevant genes through RNA-Seq of the cells from the two key immune organs of Atlantic salmon.

AIC had distinct cell population; the cells were less diverse than the whole leukocyte population. Other researchers were also able to isolate cells from the intestine of Atlantic salmon (Attaya et al., 2018) and gilthead seabream (Salinas et al., 2007), but they did not specifically examine adherent cells. The major phagocytes in vertebrates are neutrophils, monocytes, macrophages, mast cells, and immature dendritic cells (Robinson and Babcock, 1998). In the present study, we confirmed the phagocytic ability of AIC, which had different shapes compared with those of AKC. Unlike monocyte-derived macrophages which have mainly stretched shapes, the AIC appeared larger and rounded as shown in a mice study (Vereyken et al., 2011). Rombout et al. (1998) reported that the carp intestinal cells are diverse compared to the cells from other immunological tissues such as spleen and head kidney. In addition to many round-shaped cells, the oval shaped phagocytes, similar to the ones we observed, are reported as endothelial cells in mammals, and they were shown to have the ability to internalize pathogenic bacteria (Rengarajan et al., 2016). Furthermore, Lindell et al. (2012) showed that oval-shaped skin epithelial cells in trout can perform phagocytosis of *Vibrio anguillarum*. However, their identity has to be validated by employing specific cell markers. As for the doublets in the adherent cells, the phagocytes in them resembled macrophage-like cells (R1, Figure 1A) while the interacting small cells had a morphology similar to that of lymphocytes. A study that investigated mouse intestinal cells using imaging flow cytometry (Zhao et al., 2014) indicated that similar doublets consisted of CD103^+^ intestinal dendritic cells and CD4^+^ T cells. From our results, we infer that the oval-shaped cells similar to endothelial cells or epithelial cells with phagocytic ability may have special roles in mucosal immune system. In addition, the presence of the doublets indicates the interaction between phagocytes and lymphocyte-like cells that cooperate with them in immune defense.

In the present study, considering the phagocytic ability and high expression levels of macrophage genes in AIC we presume that majority of cell types in AIC are macrophages. Salmon macrophages can be effectively harvested after culturing adherent cells from HK (Paulsen et al., 2001). The presence of macrophages in adherent cells from fish intestine and their phagocytic properties have not been reported yet. In the present study, high levels of TNF-related genes (*tnfrsf10a*, *tnfsf11*, *traf2* and *tnfsf6b*) and TGFβ receptors (*tgfbr1* and *tgfbr2*) in AIC can be linked to activation and proliferation of macrophages (Luckett-Chastain et al., 2016; Yu et al., 2017) and T cells (McKarns and Schwartz, 2005; Mehta et al., 2018) while high levels of *il1* and *il6* in AKC can be linked to the classical activation of macrophages (Luckett-Chastain et al., 2016). We have also found that the expression of eight macrophage-related genes (*h2-eb1*, *cd74*, *cd68*, *marco*, *capg*, *mpeg1*, *cd200r1*, and *csf1r*) was highest in AIC among selected cell type linked genes (Figure 9) although the expression in AKC was significantly higher than those of AIC (Figure 5). Our results are in agreement with another HK transcriptomic study; carp HK macrophage-like cells had higher expression of *cd68* and *mcsfr* compared to those of HK leukocytes (Hu et al., 2018). Furthermore, a study conducted by Jenberie et al. (2018) revealed the higher expressions of *csf1r* and *marco* in HK macrophage-like cells. Another salmon study indicated the higher expression of MHCIIβ in the adherent cells from the head kidney (Iliev et al., 2019), and we found that the expression of *h2-eb1* gene was higher in AKC. This gene in association with MHC class II plays a role in the processing and presentation of antigens (Adamus et al., 2012), and teleost mucosa has major T cells receptors (TCR) such as TCRαβ and TCRγδ, and their respective co-receptors, namely CD4 and CD8, which facilitate antigen recognition (Toda et al., 2011). The presence of T cells in fish intestine was reported years ago by Rombout et al. (1998). Another study (Romano et al., 2007) also indicated the abundance of T cells and TCRβ^+^ lymphocytes in the posterior intestine and mid-intestine of seabass, respectively. In addition, CD3ε^+^ cells are abundant in salmonid immune organs including intestine (Koppang et al., 2010). Our results about the higher expression of T cell-related genes and *h2-eb1* in AIC and the doublets could be pointing to the cooperation between the adherent cell populations. AIC had higher expression of chemokines, probably those related to T cells. Furthermore, three chemokines (*ccr9l*, *ccl20l*, and *ccr6l*) were dominantly expressed in AIC. It has been reported that mammalian CD4^+^ T cells express *ccr9* (Cosorich et al., 2019) while *ccl20* and *ccr6* are expressed on CD8^+^ T cell (Kondo et al., 2007; Kish, 2015). The chemokines that are highly expressed in AKC are related to macrophage activation: teleost macrophage-like cells express *cxcr3l* (Lu et al., 2017), *ccl25* (Aquilino et al., 2016) and *cxcr4l* (Chia et al., 2018) while mammalian macrophages express *ccr3* (Park et al., 1999). In present study, higher expression levels of *ccr9l*, *ccl20l*, and *ccr6l* may be indicating the presence of T cells in AIC. Furthermore, considering our hypothesis that the adherent cells could be mainly phagocytes, the expression of T cell-related genes in the intestine could be suggestive of their critical role in mucosal immune system. A study on zebrafish (Wan et al., 2017) showed that γδ T cells which are generally abundant in the intestinal epithelium have potent phagocytic ability. In addition, the phagocytic activity of AIC and the finding of a previous study on the ability of oval-shaped mammalian endothelial cells to internalize pathogens (Rengarajan et al., 2016) likely indicate the phagocytic property of endothelial cells in AIC. We found that AIC had significantly higher expression of endothelial genes (*ecscr* and *cd151l*) while AKC had higher expression of *pecam* gene. Both *ecscr* and *cd151* are known as general signatures of mammalian endothelial cells (Verma et al., 2010; Yang et al., 2017) though they have not yet been reported in fish. On the other hand, *pecam* (or *cd31*) is a gene associated with endothelial cell adhesion molecule and it was highly expressed within blood vascular compartment (Privratsky and Newman, 2014). Furthermore, the presence of the mucin-like receptor, *cd164* could be an indication of the ability of certain cells in AIC to adhere to endothelial cells (Havens et al., 2006).

In summary, employing IFC and transcriptomics, we were able to characterize the adherent cell populations from DI, based on the genes reported as specific to certain cell types. AIC had different-shaped phagocytes and expressed genes associated with macrophages, T cells, and endothelial-like cells. Overall (selected) gene profiles show that AIC predominantly express macrophage-related genes. Further investigation on the transcriptomic responses of the intestinal cells to different antigens will help to expand our understanding of their crosstalk at the mucosal surfaces in teleosts.

## Data Availability Statement

The obtained raw sequencing data was deposited in the Gene Expression Omnibus (GEO, NCBI); the accession number is GSE154142.

## Ethics Statement

The animal study was reviewed and approved by National Animal Research Authority in Norway (Mattilsynet; FOTS ID 10050).

## Author Contributions

YP, JF, and VK conceived and designed the study. YP performed the experiments on the cells and wrote the first draft of the manuscript. YP and QZ conducted RNA sequencing and data analysis. GW provided suggestions to improve the manuscript. YP, QZ, GW, JF, and VK read, revised and approved the manuscript for submission. All authors contributed to the article and approved the submitted version.

## Conflict of Interest

The authors declare that the research was conducted in the absence of any commercial or financial relationships that could be construed as a potential conflict of interest.

## Footnotes

1 https://www.ncbi.nlm.nih.gov/assembly/GCF_000233375.1/

## Supplementary Material

Supplementary Figure 1 Dispersion estimation plot. The plot indicates the shrinkage of the gene-wise dispersions.

Supplementary Table 1 Details of raw reads, cleaned reads and mapped reads from different samples.
