# Supplementary figures and images for: Adherent Intestinal Cells From Atlantic Salmon Show Phagocytic Ability and Express Macrophage-Specific Genes
Source: Front Cell Dev Biol. 2020 Oct 15;8:580848. doi: 10.3389/fcell.2020.580848 (PMC7593592; doi:10.3389/fcell.2020.580848)

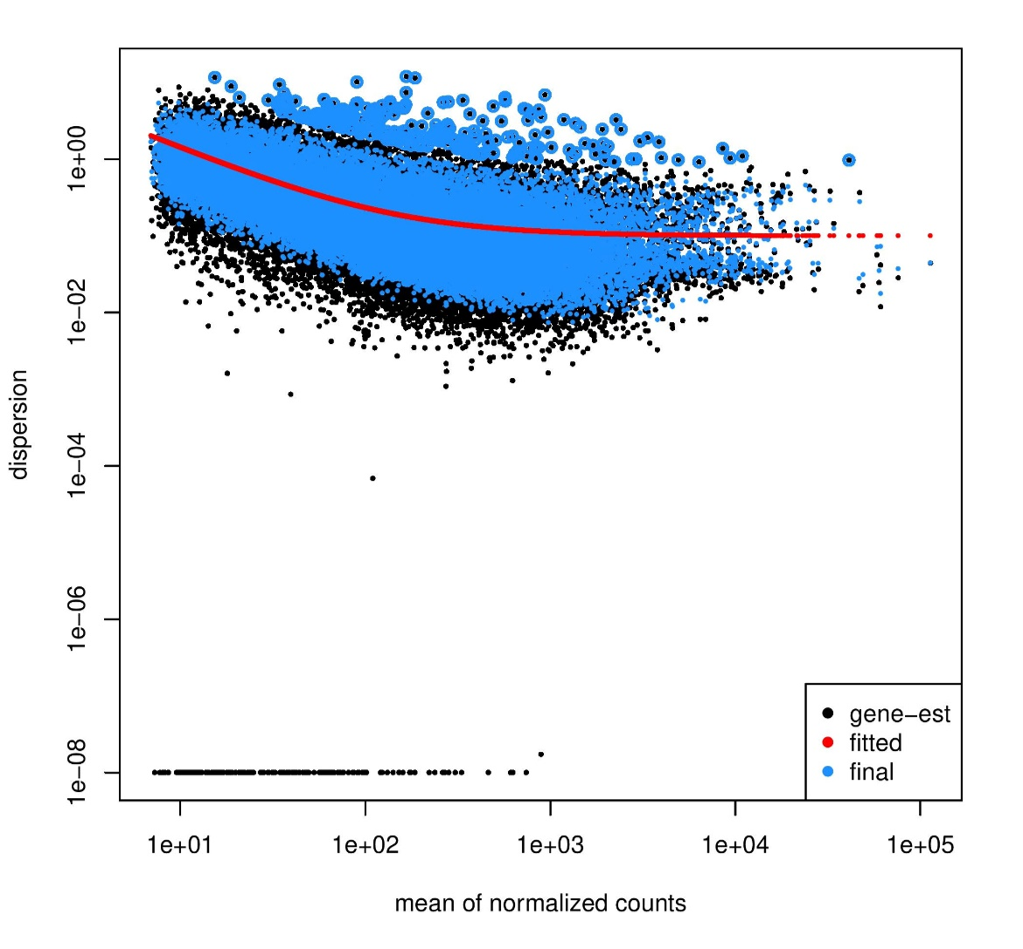

Supplement: Supplementary Figure 1 — Dispersion estimation plot. The plot indicates the shrinkage of the gene-wise dispersions. [file Image_1.TIF]
