# Supplementary material for: Adherent Intestinal Cells From Atlantic Salmon Show Phagocytic Ability and Express Macrophage-Specific Genes
Source: Front Cell Dev Biol. 2020 Oct 15;8:580848. doi: 10.3389/fcell.2020.580848 (PMC7593592; doi:10.3389/fcell.2020.580848)
Supplement: Supplementary Table 1 — Details of raw reads, cleaned reads and mapped reads from different samples. [file Table_1.DOCX]

**Supplementary Table 1.** Details of raw reads, cleaned reads and mapped reads from different samples.

| Tissue | Groups | Sample codes | Raw reads | Cleaned reads | Mapped reads | Mapped % |
| --- | --- | --- | --- | --- | --- | --- |
| Distal intestine | AIC | AIC1 | 27046689 | 24921019 | 21791819 | 87.44 |
| Distal intestine | AIC | AIC2 | 28155880 | 26696723 | 23822206 | 89.23 |
| Distal intestine | AIC | AIC3 | 27215841 | 25539454 | 23760549 | 93.03 |
| Distal intestine | AIC | AIC4 | 28333606 | 26656782 | 23259758 | 87.26 |
| Distal intestine | AIC | AIC5 | 20391222 | 19171852 | 15966073 | 83.28 |
| Distal intestine | AIC | AIC6 | 26146991 | 24906092 | 22145409 | 88.92 |
| Head kidney | AKC | AKC1 | 28423671 | 27076668 | 25805582 | 95.31 |
| Head kidney | AKC | AKC2 | 31886966 | 30270470 | 28811154 | 95.18 |
| Head kidney | AKC | AKC3 | 25729065 | 23546461 | 22270929 | 94.58 |
| Head kidney | AKC | AKC4 | 32295403 | 30236816 | 28374577 | 93.84 |
| Head kidney | AKC | AKC5 | 27779635 | 25944710 | 24714119 | 95.26 |
| Head kidney | AKC | AKC6 | 28996342 | 27168763 | 25270377 | 93.01 |

AIC: Adherent cells from distal intestine, AKC: Adherent cells from head kidney
